# Oxygenation strategies during flexible bronchoscopy: a review of the literature

**DOI:** 10.1186/s12931-021-01846-1

**Published:** 2021-09-25

**Authors:** Corrado Pelaia, Andrea Bruni, Eugenio Garofalo, Serena Rovida, Eugenio Arrighi, Gianmaria Cammarota, Paolo Navalesi, Girolamo Pelaia, Federico Longhini

**Affiliations:** 1 Pulmonary Medicine Unit, Department of Health Sciences, “Magna Graecia” University, Catanzaro, Italy; 2 Anesthesia and Intensive Care Unit, Department of Medical and Surgical Sciences, “Mater Domini” University Hospital, “Magna Graecia” University, Viale Europa, 88100 Catanzaro, Italy; 3 Department of Emergency Medicine, Royal London Hospital, Barts Health NHS Trust, London, UK; 4 Anesthesia and General Intensive Care, “Maggiore Della Carità” University Hospital, Novara, Italy; 5 Department of Medicine-DIMED, Anesthesia and Intensive Care, Padua Hospital, University of Padua, Padua, Italy

**Keywords:** Bronchoscopy, Oxygen, High flow nasal cannula, Continuous positive airway pressure, Non-invasive ventilation

## Abstract

During flexible fiberoptic bronchoscopy (FOB) the arterial partial pressure of oxygen can drop, increasing the risk for respiratory failure. To avoid desaturation episodes during the procedure several oxygenation strategies have been proposed, including conventional oxygen therapy (COT), high flow nasal cannula (HFNC), continuous positive airway pressure (CPAP) and non-invasive ventilation (NIV). By a review of the current literature, we merely describe the clinical practice of oxygen therapies during FOB. We also conducted a pooled data analysis with respect to oxygenation outcomes, comparing HFNC with COT and NIV, separately. COT showed its benefits in patients undergoing FOB for broncho-alveolar lavage (BAL) or brushing for cytology, in those with peripheral arterial oxyhemoglobin saturation < 93% prior to the procedure or affected by obstructive disorder. HFNC is preferable over COT in patients with mild to moderate acute respiratory failure (ARF) undergoing FOB, by improving oxygen saturation and decreasing the episodes of desaturation. On the opposite, CPAP and NIV guarantee improved oxygenation outcomes as compared to HFNC, and they should be preferred in patients with more severe hypoxemic ARF during FOB.

**Supplementary Information:**

The online version contains supplementary material available at 10.1186/s12931-021-01846-1.

## Introduction

Flexible fiberoptic bronchoscopy (FOB) is a diagnostic and sometimes therapeutic procedure, commonly performed in patients affected by airway or lung parenchyma disorders. FOB has several applications, including plug removal in presence of abundant secretions or ineffective cough, bronchoalveolar lavage (BAL), biopsy, or endoscopic management of bleeding.

The majority of patients undergoing FOB suffer from conditions that impair gas exchange such as pneumonia, interstitial lung diseases, as well as lung and bronchial neoplasms. During the procedure arterial partial pressure of oxygen can drop even more than 10–20 mmHg, with an increased risk for respiratory failure [1, 2]. In order to avoid desaturation episodes, oxygen support provided by conventional therapy or non-invasive ventilation is usually required during and after FOB.

Through a review of the literature, we discuss the rationale and all the alternative oxygenation strategies adopted during FOB. In addition, in the attempt to provide some clinical evidences, we have also conducted a quantitative synthesis of findings comparing high flow oxygen through nasal cannula (HFNC) with conventional oxygen therapy (COT) and non-invasive ventilation (NIV), separately, with respect to the lowest saturation during procedures and the number of episodes of desaturation.

## Evidence acquisition

This review was conducted in accordance with the Preferred Reporting Items for Systematic reviews and Meta-Analyses (PRISMA) statement. The review protocol has been registered in Prospero (CRD42020153343).

### Study selection and inclusion criteria

All cited articles include adult patients, receiving one or more modalities of oxygen support administered during flexible bronchoscopy for any reason (diagnostic or interventional), without restrictions related to the type of bronchoscopy procedure and to the anesthetic risk.

We included all randomized, quasi-randomized, prospective and retrospective studies, published in indexed scientific journals from inception to May 1st, 2021. We excluded papers published in languages other than English, Italian, French or Spanish as well as case reports or series, review, systematic reviews or meta-analysis and studies published in abstract form. Papers including patients undergoing rigid bronchoscopy were also excluded. References of included papers, reviews, systematic reviews and meta-analysis were also examined to identify potential studies of interest missed during the primary search.

All oxygen therapy modalities utilized during flexible bronchoscopy were evaluated. Specifically, we considered: (1) COT, consisting of low oxygen flow administration through nasal prongs, oxygen mask with or without reservoir, and Venturi mask [3]; (2) HFNC, consisting of administration of high flows (up to 60 L/min) of air/oxygen admixtures, heated (at temperatures ranging from 31 to 37 °C) and fully humidified (up to 44 mg H_2_O/L) [4], providing an inspired oxygen fraction ranging from 21 to 100%; (3) continuous positive airway pressure (CPAP), based on the application of a positive end-expiratory pressure (PEEP) throughout the whole respiratory cycle by means of interfaces such as mask or helmet [5, 6], and (4) NIV, based on the application of a PEEP by means of a mask or helmet, with an inspiratory pressure support triggered by the patient and delivered by a ventilator [7, 8].

### Search strategy

Two authors (A.B. and C.P.) independently searched MEDLINE, EMBASE, and Scopus Database of Systematic Reviews using the following keywords and their related MeSH terms: "bronchoscopy", "conventional oxygen therapy", "continuous positive airway pressure", "bilevel continuous positive airway pressure", "airway pressure release ventilation", "noninvasive ventilation", and "high flow nasal oxygen". The search strategy is detailed in the Electronic Supplemental Material (ESM). Controlled vocabulary terms, text words, and keywords were variably combined. Blocks of terms per concept were created. These authors also independently checked all the articles, and selected those enrolling adult patients undergoing bronchoscopy which required oxygen therapy or other modalities of respiratory support. In case of disagreement, the opinion of a third examiner (F.L.) was requested for a conclusive decision.

### Definition of clinical outcomes

A quantitative synthesis of findings has been conducted for the lowest saturation during procedures and the number of episodes of desaturation. The lowest saturation was defined as the lowest value reported by the included studies of the arterial (SaO_2_) or peripheral (SpO_2_) oxygen saturation during the FOB procedure. The number of episodes of desaturation was intended as the number of patients with one or more episodes of desaturations during the procedure, as defined by SaO_2_ or SpO_2_ < 90% for a minimum time defined by every single study.

### Statistical analysis

Dichotomous outcomes are presented as risk ratios (RR) with 95% confidence intervals (CIs). For normally distributed continuous data, we have calculated the mean difference (MD) with corresponding 95% CIs. We use medians and interquartile ranges for continuous data that were not normally distributed. Meta‐analyses have been performed using random‐effects models. We have assessed heterogeneity by visually inspecting the forest plots to determine closeness of point estimates with each other and overlap of CIs. We used the χ^2^ test with a P value of 0.10 to indicate statistical significance, and the I^2^ statistic to measure heterogeneity. We have also considered the magnitude and direction of effects, and the strength of evidence for heterogeneity (e.g. P value from the χ^2^ test), when determining the importance of the observed I^2^ value. P values < 0.05 were considered statistically significant.

## Results

**Fig. 1:**
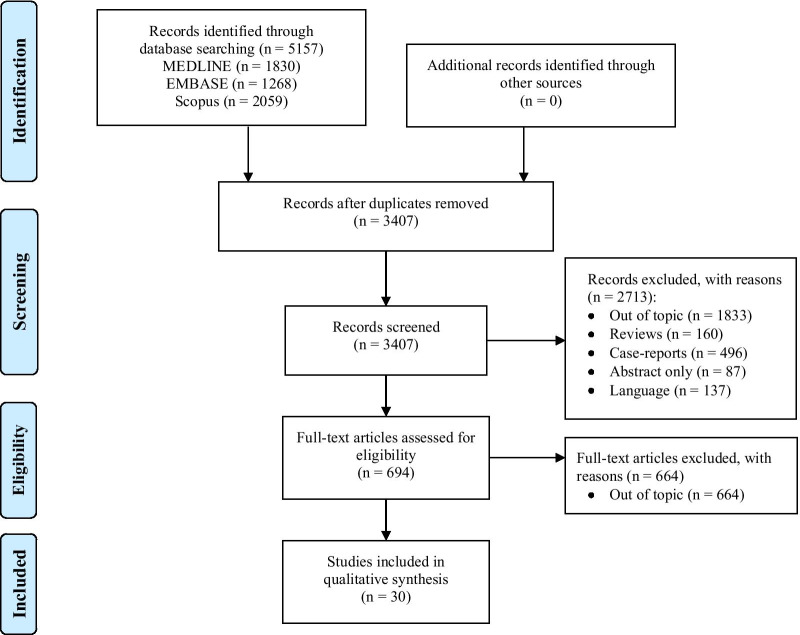
Flow chart of reviewed studies

The electronic search identified 5157 potentially relevant studies. Detailed description of the selection process flow is provided in Fig. 1. We selected 32 full-text manuscripts (Table 1), referring to 3 multi-centered and 29 single-centered studies, respectively [1, 9–39]. With the exception of 7 studies, all trials were performed in University Hospitals. Among all studies, 18 were conducted in European countries. Overall, the 32 selected studies enrolled 2517 patients with a median of 40 [12–60] patients per study, and a median patient age of 60 [53–64] years.

**Table 1:** Characteristics of the studies included in the systematic review

	Year	Patients enrolled	FOB performed	Study type	Clinical status	Setting	RS before FOB	RS during FOB	FOB purpose	Analgosedation strategy	Main outcomes
Antonelli M., et al. [1]	1996	8	8	Interventional	Acute	ICU	COT	NPPV	Diagnostic	Lidocaine	O_2_ saturation, PaO_2_/FiO_2_, PaCO_2_, RR, HR, intubation, mortality
Jones A.M., et al. [9]	2000	1074	2261	Observational	Chronic	Ward	None	COT	Diagnostic	Midazolam, lignocaine	O_2_ saturation
Onakpoya U.U., et al. [10]	2012	61	67	Observational	Chronic	Ward	None	COT	Diagnostic	Diazepam, lignocaine	O_2_ saturation
Golpe R., Mateos A. [11]	2002	44	44	Observational	ND	ND	None	COT	ND	Lignocaine	O_2_ saturation
Alijanpour E., et al. [12]	2010	146	146	Observational	ND	ND	None	COT	ND	Lidocaine	O_2_ saturation
Cracco C., et al. [13]	2013	169	181	Observational	Acute	ICU	COT, NPPV	COT, NPPV	Diagnostic, therapeutic	ND	Intubation, increased ventilatory support
Ghio A.J., et al. [14]	2007	12	12	Prospective randomized	Healthy volunteers	Outpatients	COT or none	COT or none	Research	Lidocaine	O_2_ saturation
McCain T.W., et al. [15]	2001	97	97	Prospective randomized	Chronic	ND	COT	COT	Diagnostic	Meperidine or fentanyl, midazolam	O_2_ saturation, O_2_ flow rate
Miyagi K., et al. [16]	2014	5	5	Observational	Acute	ICU	COT	HFNC	Diagnostic	ND	O_2_ saturation
La Combe B., et al. [17]	2016	30	30	Observational	Acute	ICU	COT	HFNC	Diagnostic	ND	O_2_ saturation, dyspnea, intubation, mortality
Longhini F., et al. [18]	2021	36	36	Prospective randomized	Chronic	Outpatients	None	COT or HFNC	Diagnostic	Lidocaine	O_2_ saturation, PaO_2_, ΔEELI, DD, TF
Chung S.M., et al. [19]	2018	10	10	Observational	Acute, chronic	Ward	COT	HFNC	Diagnostic, therapeutic	Midazolam, propofol, lidocaine	O_2_ saturation
Service J.A., et al. [20]	2018	182	182	Observational	Chronic	Outpatients	None	HFNC	Diagnostic	Remifentanil. propofol or midazolam	O_2_ saturation
Ben-Menachem E., et al. [21]	2020	76	76	Prospective randomized	Chronic	Ward	None	COT or HFNC	Diagnostic	ND	O_2_ saturation
Lucangelo U. et al. [22]	2012	45	45	Prospective randomized	Acute, chronic	Ward	None	COT or HFNC	Diagnostic	Midazolam, lidocaine	O_2_ saturation, PaO_2_, PaO_2_/FiO_2_
Irfan M., et al. [23]	2021	40	40	Prospective randomized	Chronic	Ward	None	COT or HFNC	Diagnostic	ND	O_2_ saturation
Douglas N., et al. [24]	2018	60	60	Prospective randomized	Chronic	ND	None	COT or HFNC	Diagnostic	Midazolam, opioids and/or propofol, lidocaine	O_2_ saturation
Kim E.J., et al. [25]	2018	32	33	Observational	Acute	Ward	COT or HFNC	HFNC	Diagnostic	Midazolam, lidocaine	O_2_ saturation PaO_2_/FiO_2_
Maitre B., et al. [26]	2000	30	30	Prospective randomized	Acute	Ward, ICU	COT	COT or CPAP	Diagnostic	Lidocaine	O_2_ saturation, PaO_2_, ventilatory assistance requirement
Antonelli M., et al. [27]	2002	26	26	Prospective randomized	Acute	ICU	COT	COT or NPPV	Diagnostic	Lidocaine	PaO_2_/FiO_2_, HR, MAP, intubation
Baumann H.J., et al. [28]	2011	40	40	Observational	Acute	ICU	NIV	NIV	Diagnostic, therapeutic	Midazolam, propofol, lidocaine	O_2_ saturation, PaO_2_/FiO_2_, PaCO_2_, intubation
Korkmaz Ekren P., et al. [29]	2016	28	28	Observational	Acute	ICU	NIV	NIV	Diagnostic, therapeutic	Midazolam, lidocaine	PaO_2_/FiO_2_, intubation
Chiner E., et al. [30]	2010	35	35	Observational	Acute	Ward, ICU	NIPPV	NIPPV	Diagnostic, therapeutic	Lidocaine	O_2_ saturation, RR, HR, intubation, mortality
Heunks L.M.A., et al. [31]	2010	12	12	Observational	Acute	ICU	COT	NPPV	Diagnostic	Xylocaine	O_2_ saturation, PaO_2_/FiO_2_, intubation
Antonelli M., et al. [32]	2003	4	4	Interventional	Acute	ICU	COT	NPPV	Diagnostic	Lidocaine	O_2_ saturation, PaO_2_/FiO_2_, PaCO_2_, RR, HR, MAP, intubation
Agarwal R., et al. [33]	2012	6	6	Observational	Acute	Ward	COT	NIV	Diagnostic, therapeutic	Lidocaine	O_2_ saturation, PaO_2_/FiO_2_, intubation
Chen X., et al. [34]	2020	29	29	Retrospective	Acute	Ward	COT	NIV, CMV	Therapeutic	Dezocine, midazolam, lidocaine	O_2_ saturation, PaO_2_, HR, SBP, type of procedures, success rate, procedure time
Da Conceiçao M., et al. [35]	2000	10	10	Prospective	Acute	ICU	None	NIPPV	Diagnostic	Lidocaine	O_2_ saturation, PaO_2_, PaCO_2_, intubation, mortality
Mohamed A.S., El-Sharawy D.E. [36]	2018	40	20	Prospective randomized	Acute	Ward	NIV	NIV	Therapeutic	Midazolam, lidocaine	O_2_ saturation
Scala R., et al. [37]	2010	30	30	Prospective	Acute	RICU, ICU	NPPV	NPPV, CMV	Diagnostic	Lidocaine	O_2_ saturation, PaO_2_/FiO_2_, PaCO_2_, pH
Simon M., et al. [38]	2014	40	40	Prospective randomized	Acute	ICU	COT	NIV or HFNC	Diagnostic, therapeutic	Propofol, lidocaine	O_2_ saturation, PaO_2_/FiO_2_, PaCO_2_, HR, MAP, procedure time, intubation, mortality
Saksitthichok B., et al. [39]	2019	51	51	Prospective randomized	Acute	Ward or RICU	NIV or HFNC	NIV or HFNC	Diagnostic	Fentanyl, lidocaine	O_2_ saturation, PaO_2_, PaCO_2_, pH, RR, HR, MAP, dyspnea

*ND* not defined, *FOB* flexible fiberoptic bronchoscopy, *RS* respiratory support, *ICU* intensive care unit, *RICU* respiratory intermediate care unit, *NIV* noninvasive ventilation, *COT* conventional oxygen therapy, *CPAP* continuous positive airway pressure, *HFNC* high flow oxygen through nasal cannula, *CMV* conventional mechanical ventilation, *PaO*_*2*_ arterial partial pressure of oxygen, *FiO*_*2*_ fraction of inspired oxygen, *PaCO*_*2*_ arterial partial pressure of carbon dioxide, *RR* respiratory rate, *HR* heart rate, *ΔEELI* changes of end-expiratory lung impedance, *DD* diaphragm displacement, *TF* thickening fraction, *MAP* mean arterial pressure, *SBP* systolic blood pressure

### Different forms of oxygen therapies and ventilatory support: the choice rationale

Alteration of respiratory mechanics during FOB occurs physiologically. In non-intubated patients, the fiberscope occupies about 10% of the cross-sectional area of the trachea, and 15% of the cricoid ring. As a consequence, in patient's airways an increase in air flow resistance develops, and the work of breathing thus enhances [40]. When suction is applied, end-expiratory lung volume is reduced, leading to alveolar de-recruitment, increased shunt and venous admixture [40–42]. As mentioned, these respiratory changes revert after FOB, but their reversal may take from 15 min up to several hours in severe parenchymal lung diseases [40].

In addition, FOB indirectly causes also significant haemodynamic changes. The increase of airway resistance and work of breathing theoretically leads to changes in intra-thoracic pressure, that may also affect venous return and afterload, while reducing cardiac output. However, it has been reported that cardiac output increases by 50% secondary to sympathetic stimulation during FOB, and it returns to baseline in 15 min after its completion [40, 43, 44]. In fragile or cardiopathic patients, FOB may cause a dangerous cardiopulmonary distress, associated with electrocardiographic alteration in up to 21% of awake patients over 60 years old [44]. This aspect should also drive the clinician to choose the more appropriate oxygenation strategy from a physiological point of view.

COT through nasal prongs is appropriate to reduce transitory hypoxemia [42]. However, the inspired oxygen fraction (FiO_2_) cannot be predicted, and might not be enough in severe cases.

When lung parenchyma is already compromised by an underlying pathologic condition, uncomplicated FOB may worsen gas exchange, leading to development of respiratory failure [1, 2, 41, 45]. HFNC has been introduced in clinical practice as a valuable alternative for oxygen support during FOB [4, 46–48]. Four main reasons support its use during and after the procedure: (1) the flow up to 60 L/min ensures a more stable FiO_2_, as it is able to match the increased patient’s inspiratory flow [49]; (2) the high flow generates a small positive expiratory airway pressure depending on the flow rate, the upper airway anatomy, the breathing through the nose or mouth, and the size of the cannula in relation to the nostrils [49]; (3) the high flow reduces the dead space in the upper airways up to 70 ml, and increases the alveolar ventilation [49]; and (4) the HFNC decreases the resistive breathing effort, reducing the upper airway resistance [49].

The application of CPAP has also been adopted during FOB. CPAP releases a positive pressure throughout the entire breathing cycle, thereby recruiting lung atelectatic regions [50], reducing venous admixture [51], and decreasing the patient’s inspiratory effort [5].

The use of NIV can also ameliorate gas exchange, thus diminishing the respiratory effort. However, despite these benefits NIV is negatively affected by poor patient-ventilator interaction, which impairs its effectiveness [7, 8, 52–54].

### COT during FOB

The use of COT during FOB has been investigated in several studies. Although not always required [9], COT showed its benefits in patients with higher risk of desaturation, including those with baseline (before procedure) peripheral arterial oxyhemoglobin saturation (SpO_2_) < 93% [10], an obstructive ventilatory defect [11], or a forced expiratory volume in the first second (FEV_1_) lower than 1 L [9, 12]. COT is also indicated in those patients suffering from chronic obstructive pulmonary disease and immunosuppression, due to their higher risk of intubation in the 24 h following FOB [13].

When FOB is carried out for BAL [14] or brushing performed for cytological examination [10], COT is needed as well. Oxygen can be delivered by a cannula placed either in the nose or in the mouth, as the average SpO_2_ within the procedure is similar between the two modalities, and no sinus symptoms or nasal congestion have been reported [15].

### HFNC during FOB

HFNC consists of a mixture of air and oxygen, delivered through a maximally heated and humidified flow up to 60 L/min.

HFNC has been successfully used to prevent acute respiratory failure (ARF) from worsening during FOB. A small study conducted on 5 patients found that oxygenation was well maintained for 30 min after FOB for BAL, and only one patient required non-invasive positive pressure ventilation 16 h after FOB [16]. Similar results were reported in regard to a larger observational trial, with 30 critically ill patients affected by ARF during nasal FOB performed with non-open mouth [17] and 60 patients requiring FOB for BAL [18]. Improvement of post-FOB SpO_2_ was also described, as well as prevention of mucosal injury and patient discomfort, thanks to the humidified and heated gas flow [19]. In a prospective study carried out using HFNC during FOB for endobronchial ultrasound (EBUS) with deep sedation, no difference in desaturation events was detected between procedures lasting less or more than 40 min [20]. In several studies, HFNC has also been compared with other oxygen support modalities, as shown below. In a randomized controlled trial on post-lung transplant patients undergoing FOB for transbronchial lung biopsy, the procedure was interrupted when low-flow nasal oxygen was applied, whereas no similar episodes occurred with HFNC [21]. When compared to Venturi mask, HFNC at 60 L/min provided better oxygenation outcomes, whereas no difference was observed between HFNC 40 L/min and Venturi mask 40 L/min [22]. A very recent randomized controlled trial showed that the use of HFNC instead of COT, during FOB for EBUS, was associated with a statistically significant lower drop in SpO_2_ [23].

When compared with COT in patients without ARF undergoing EBUS with conscious sedation, HFNC did not significantly reduce the rate of intraprocedural desaturation episodes [24]. During FOB for BAL, effectiveness and safety of HFNC versus COT was tested in patients with ARF [25]. No relevant events, such as endotracheal intubation, were reported within the 24 h following FOB for BAL. No statistically significant differences between patients undergoing or not HFNC with regard to transient hypoxemia, fever, hypotensive events, and endotracheal intubation rate were observed [25].

### CPAP during FOB

The use of CPAP through full-face mask during FOB has been investigated in patients with hypoxemic ARF (PaO_2_/FiO_2_ < 300 mmHg) by Maitre et al. [26]. As compared to COT, CPAP guaranteed higher SpO_2_ during and after the procedure, and lower numbers of patients required ventilatory assistance within the 6 h following the endoscopic procedure [26].

### NIV during FOB

NIV via face mask was for the first time applied during FOB for BAL in 1996 [1]. In a cohort of immunosuppressed patients with suspected pneumonia and severe hypoxemia (PaO_2_/FiO_2_ < 100 mmHg), Antonelli et al. reported that NIV, applied 10 min before and discontinued 90 min after FOB, was well tolerated by all patients, improved gas exchange, and prevented the need for intubation [1].

Later on, the application of NIV via face mask was also evaluated in immunocompetent patients with hypoxemic ARF of different severity. NIV provided optimal gas exchange during and after FOB [27–29], without causing hemodynamic impairments [27], and was also associated with a low incidence of minor complications [29], and with a small percentage of patients requiring intubation during the first 8 h after the procedure [28, 29].

At that time, facial masks were adapted with swivel connectors [28] or sealed ports [27] for the passage of the bronchoscope into the interface and patients’ airways. Nasal masks have been proposed as a valid alternative to facial masks for NIV during FOB [30]. In addition, new masks for NIV specifically designed for FOB were also tested [29–31]. Lastly, helmet NIV represents another feasible and safe alternative to NIV via mask during FOB for BAL in patients with hypoxemic ARF [32].

The use of NIV during FOB has been also extended to more complicated procedures, such as transbronchial lung biopsy [33] and interventional procedures, including balloon dilation, electrocautery and argon plasma coagulation [34]. In keeping with the above-mentioned studies, NIV guaranteed a stable oxygenation [33, 34] and good patients’ tolerance [33], whereas minor complications [34], as well as the number of patients requiring intubation after the procedure [33], were quite low. Similar findings have been also reported for patients with chronic [35] and acute-on-chronic [36, 37] respiratory failure.

The growing evidence in favor of both HFNC and NIV has yielded some studies comparing the two modalities in patients with mild-to-moderate hypoxemic ARF. Compared to HFNC, NIV improved oxygenation before, during and after FOB [38, 39], as well as decreased the number of desaturations < 90% [39], without any difference in mortality or in the rate of patients requiring intubation and invasive mechanical ventilation [38, 39].

### Quantitative synthesis of study findings

**Fig. 2:**
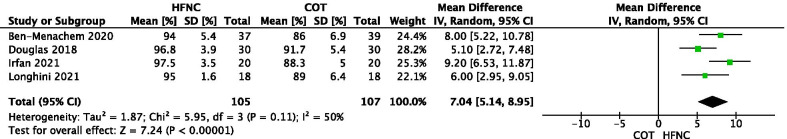
Quantitative synthesis of HFNC versus COT with respect to the lowest saturation

**Fig. 3:**
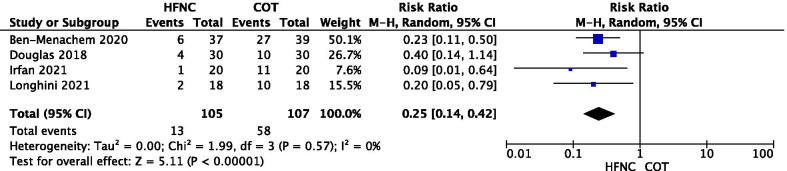
Quantitative synthesis of HFNC versus COT with respect to the episodes of desaturation during the FOB

Figures 2 and 3 depict the quantitative synthesis of HFNC versus COT with respect to the lowest saturation and the episodes of desaturation during the FOB, respectively. Funnel plots for visual inspection of heterogeneity are included in the Additional file 1: Figures S1 and S2, respectively. In comparison with COT, HFNC significantly improves the lowest saturation (MD 7.04 [95%CI: 5.14 to 8.95]%; p < 0.001; I^2^ = 50%) and it significantly reduces the number of episodes of desaturation (RR 0.25 [95%CI: 0.14 to 0.42]; p < 0.001; I^2^ = 0%).

**Fig. 4:**
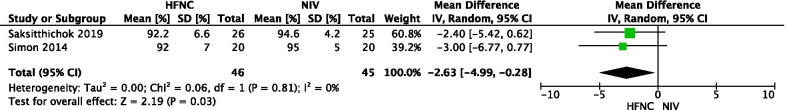
Quantitative synthesis of HFNC versus NIV with respect to the lowest saturation

**Fig. 5:**
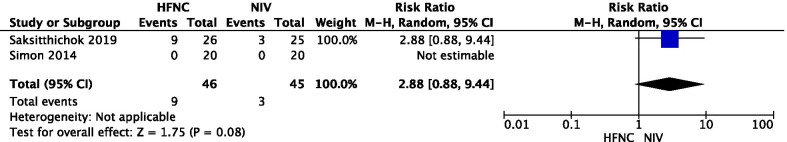
Quantitative synthesis of HFNC versus NIV with respect to the episodes of desaturation during the FOB

In addition, Figs. 4 and 5 depict the quantitative synthesis of HFNC versus NIV with respect to the lowest saturation and the episodes of desaturation during the FOB, respectively. Funnel plots for visual inspection of heterogeneity are also included in the Additional file 1: Figures S3 and S4, respectively. As opposed to NIV, HFNC is characterized by a reduced lowest saturation (MD − 2.63 [95%CI: − 4.99 to − 0.28]%; p = 0.03; I^2^ = 0%). On the opposite, HFNC and NIV do not differ with respect to the number of episodes of desaturation (RR 2.88 [95%CI: 0.88 to 9.44]; p = 0.08; I^2^ = not applicable). Noteworthy, these data are reported only in the study by Simon et al. [38].

## Discussion

Although several studies showed benefits from different oxygenation strategies during FOB, no clear guidelines are yet available in literature. Overall, the worst is the patient's baseline lung function prior to the procedure, the highest is the oxygen requirement within the procedure and the risk of worsening ARF afterwards.

HFNC was shown to be safe in the majority of patients affected by mild-to-moderate ARF undergoing FOB for either diagnosis or treatment, and in those with lung transplant, while NIV ensured stable oxygenation when FOB was carried out for extended procedures or in patients with more severe ARF. However, information is still very scarce about the eventual better advantages of one strategy compared to another. All patients’ categories mentioned in the reviewed studies, as well as the oxygenation modalities which resulted more successful, have been summarized in Table 2. Generally speaking, by the pooled data analysis, HFNC outperforms COT with respect to oxygenation outcomes in patients with lower oxygen requirement, whereas data suggest the superiority of NIV in patients with more severe ARF, as compared to HFNC. Although the lack of sufficient evidence prevents the possibility to provide a clear or definitive recommendation on the use of an oxygenation strategy over another one, the oxygenation improvement during the procedure still remains an important safety issue for patients undergoing FOB. In addition, it potentially may improve major clinical outcomes (such as the need for hospital or ICU admission for post-procedural respiratory failure); however, such benefits require to be addressed.

**Table 2:** The table summarizes the oxygen modalities that have been adopted according to the underlying patient’s lung disorder and FOB indications current literature

Indications	Respiratory conditions					
	FEV_1_ < 1 L	COPD	Immunodepression	Hypoxemic ARF (PaO_2_/FiO_2_ > 200)	Hypoxemic ARF (PaO_2_/FiO_2_ < 200)	Lung Tx
BAL	COT	COT/HFNC	COT/HFNC	HFNC	CPAP/NIV	
Brushing for cytology		COT	COT	HFNC	CPAP/NIV	
EBUS deep sedation		HFNC	HFNC	HFNC	CPAP/NIV	
EBUS conscious sedation		COT/HFNC	COT/HFNC	HFNC	CPAP/NIV	
Lung biopsy						HFNC
Balloon dilatation Electrocautery APC	NIV	NIV			NIV	

*BAL* bronchoalveolar lavage, *EBUS* endobronchial ultrasound, *APC* argon plasma coagulation, *SpO*_*2*_ peripheral arterial oxyhemoglobin saturation, *FEV*_*1*_ forced expiratory volume in the first second, *COPD* chronic obstructive pulmonary disease, *ARF* acute respiratory failure, *PaO*_*2*_*/FiO*_*2*_ ratio between arterial partial pressure to inspired fraction of oxygen, *Tx* transplantation, *COT* conventional oxygen therapy, *HFNC* high flow oxygen through the nasal cannula, *CPAP* continuous positive airway pressure, *NIV* noninvasive ventilation

In such a heterogeneous scenario of lung conditions, and with FOB implicated in a variety of diagnostic and therapeutic procedures, a score able to predict the occurrence of adverse events could support the clinician when deciding the best oxygenation modality. In order to develop such a score, a relevant scientific effort based on a multicenter research study would be useful.

The choice of oxygenation strategy may interfere with the route of access of the FOB. For example, during HFNC the presence of large bore nasal prongs prevents the possibility to use the nasal route, leading the physician to insert the FOB through the mouth. Noteworthy, the small positive expiratory airway pressure generated by HFNC would be significantly reduced during open mouth breathing, loosing theoretically its benefit on alveolar distending pressure and lung de-recruitment prevention [49]. However, we have recently demonstrated that in outpatients undergoing FOB with BAL, when compared to COT, HFNC prevents oxygenation worsening by avoiding end-expiratory loss of lung volume and preserves the same tidal volume with a lower diaphragm activation [18]. Therefore, based on our experience and recent data [18], we suggest the use of HFNC, rather than COT, in out-patients undergoing FOB with BAL.

On the opposite, some interfaces (helmet and face masks) for CPAP o NIV have been specifically designed to be used during FOB, which allow both oral and nasal route for bronchoscope insertion. For example, Korkmaz Ekren et al. have used a dedicated full-face mask with an interchangeable connector between the ventilator tubing and the mask that allows the insertion of the bronchoscope through a sealed-hole [29]. In another study by Heunks et al. [31], the investigators used a total face mask with a dedicated sealed hole below the connector between ventilator tubing and interface allowing the performance of the FOB through the mouth during NIV. However, air-leaks may occur around the interface or through the dedicated hole for FOB insertion, leading to patient-ventilator asynchronies which may be difficult to be managed [8, 55]. As a suggestion based on our experience and previous data [26], CPAP may be preferable over NIV for some reasons: first of all, CPAP is more user-friendly to be applied, as compared to NIV. In fact, the need of a simple flow generator, rather than a ventilator, makes CPAP easy to be applied. Moreover, although both CPAP and NIV guarantee the application of positive airway pressure throughout the whole respiratory cycle, the latter requires to adjust ventilator settings in order to improve the patient-ventilator synchrony during the inspiratory phase [8, 55].

The oxygenation strategy should also be chosen according to the procedure. Although some procedures (i.e. FOB with BAL) may be performed with an extensive topical anesthesia, others (i.e. EBUS) may require deeper sedation with different pharmacological strategies including both sedatives (i.e. midazolam, propofol or dexmedetomidine) and analgesics (i.e. remifentanil). However, it must be recognized that these drugs can modify the critical closing pressure of the upper airways, inducing their collapse [56, 57], and they can affect the breathing pattern and/or the respiratory drive. In particular, the deeper is the sedation, the higher is the modification [58, 59]. Therefore, in case of deep sedation, NIV (or even the placement of a laryngeal mask) may be required and preferred over other oxygenation strategies to ensure breathing and gas-exchange. A literature review and, eventually, trials focused on this topic are advisable.

Finally, in the era of the ongoing epidemic, a careful choice of oxygenation strategy should also be done in case of patients with suspected or confirmed severe acute respiratory syndrome coronavirus 2 (SARS-CoV-2) infection. FOB is considered as aerosol generating procedure generating a significant number of droplets that can be contagious for other patients and the healthcare personnel [60]. Operators should firstly check the infection by SARS-CoV-2 through molecular test with naso-pharyngeal swabs; in case of positivity, full personal protective equipment (i.e. FFP-2 masks, gloves, goggles, face shields and gowns) are required. Furthermore, when exhaled air is released into the room, the dispersion of the virus may increase the risk of infection of other patients and the healthcare personnel [61]. It is well known that different interfaces are characterized by dissimilar air dispersion distances during their application [62, 63]. In principle, the use of a helmet for CPAP or NIV with a good seal around the neck is preferable, and, as abovementioned, the appropriate use of personal protective equipment is mandatory. In addition, simple practical measurements like reducing the number of assisting personnel and cough restriction with the administration of oropharyngeal lignocaine can minimize the contamination risk [60]. Of note, masks with vent holes should be avoided and a filter between the mask and the vent or PEEP valve is advisable to reduce viral transmission [61].

To our knowledge, this is the first review outlining the oxygenation strategies during FOB, and data are updated to the last available literature sources. Despite the consistent number of cited studies, the majority of them assessed the oxygenation effects on physiological, rather than major clinical outcomes. The quality of reviewed studies is also questionable, due to the small sample size and the high population heterogeneity preventing a further meta-analysis. Moreover, most of included studies are single-centered. Finally, studies enroll patients which are not exclusively chronic or acute, but sometimes are mixed or not clear populations, preventing us to the possibility to separate the findings on different oxygenation strategies according to the clinical status of the patients. Hence, finding generalization is limited, as supported by weak evidence. Noteworthy, this review highlights the need for future research and robust data, in order to draw specific recommendations in a field where clinical practice nowadays is left to single-center experience, rather than scientific evidence.

## Conclusion

In conclusion, the oxygenation strategy during FOB should be chosen according to the procedure, lung and heart function, oxygen requirement within the procedure and the risk of worsening ARF afterwards. In patients with mild-to-moderate oxygen requirement, HFNC would be preferable over COT, while the use of CPAP or NIV is encourageable in patients with more severe hypoxemia.

## Supplementary Information

**Additional file 1: Figure S1.** Funnel plot for HFNC versus COT with respect to the lowest saturation during FOB. **Figure S2.** Funnel plot for HFNC versus COT with respect to the episodes of desaturation. **Figure S3.** Funnel plot for HFNC versus NIV with respect to the lowest saturation during FOB. **Figure S4.** Funnel plot for HFNC versus NIV with respect to the episodes of desaturation.

## Data Availability

Not applicable.

## Abbreviations

ARF: Acute respiratory failure
BAL: Broncho-alveolar lavage
COT: Conventional oxygen therapy
CPAP: Continuous positive airway pressure
EBUS: Endobronchial ultrasound
ESM: Electronic supplemental material
FEV_1_: Forced expiratory volume in the first second
FiO_2_: Inspired oxygen fraction
FOB: Fiberoptic bronchoscopy
HFNC: High flow nasal cannula
NIV: Non-invasive ventilation
PaO_2_/FiO_2_: Arterial partial pressure to inspired oxygen fraction ratio
PEEP: Positive end-expiratory pressure
SARS-CoV-2: Severe acute respiratory syndrome coronavirus 2
SpO_2_: Peripheral arterial oxyhemoglobin saturation

## References

[1] Antonelli M, Conti G, Riccioni L, Meduri GU (1996). Noninvasive positive-pressure ventilation via face mask during bronchoscopy with BAL in high-risk hypoxemic patients. Chest.

[2] Goldstein RA, Rohatgi PK, Bergofsky EH, Block ER, Daniele RP, Dantzker DR (1990). Clinical role of bronchoalveolar lavage in adults with pulmonary disease. Am Rev Respir Dis.

[3] O’Driscoll BR, Howard LS, Davison AG, British Thoracic Society (2008). BTS guideline for emergency oxygen use in adult patients. Thorax.

[4] Bruni A, Garofalo E, Cammarota G, Murabito P, Astuto M, Navalesi P (2019). High flow through nasal cannula in stable and exacerbated chronic obstructive pulmonary disease patients. Rev Recent Clin Trials.

[5] Lenique F, Habis M, Lofaso F, Dubois-Randé JL, Harf A, Brochard L (1997). Ventilatory and hemodynamic effects of continuous positive airway pressure in left heart failure. Am J Respir Crit Care Med.

[6] Bellani G, Patroniti N, Greco M, Foti G, Pesenti A (2008). The use of helmets to deliver non-invasive continuous positive airway pressure in hypoxemic acute respiratory failure. Minerva Anestesiol.

[7] Nava S, Navalesi P, Carlucci A (2009). Non-invasive ventilation. Minerva Anestesiol.

[8] Garofalo E, Bruni A, Pelaia C, Liparota L, Lombardo N, Longhini F (2018). Recognizing, quantifying and managing patient-ventilator asynchrony in invasive and noninvasive ventilation. Expert Rev Respir Med.

[9] Jones AM, O’Driscoll R (2001). Do all patients require supplemental oxygen during flexible bronchoscopy?. Chest.

[10] Onakpoya UU, Adewole O, Ogunrombi AB, Adenekan AT (2012). Oxygen supplementation during awake fibreoptic bronchoscopy in a Nigerian tertiary hospital. West Afr J Med.

[11] Golpe R, Mateos A (2002). Supplemental oxygen during flexible bronchoscopy. Chest.

[12] Alijanpour E, Nikbakhsh N, Bijani A, Baleghi M (2010). Evaluation of oxygen requirement in patients during fiberoptic bronchoscopy. Caspian J Intern Med.

[13] Cracco C, Fartoukh M, Prodanovic H, Azoulay E, Chenivesse C, Lorut C (2013). Safety of performing fiberoptic bronchoscopy in critically ill hypoxemic patients with acute respiratory failure. Intensive Care Med.

[14] Ghio A, Bassett MA, Levin D, Montilla T (2007). Oxygen supplementation is required in healthy volunteers during bronchoscopy with lavage. J Bronchol.

[15] McCain TW, Dunagan DP, Adair NE, Chin R (2001). Prospective randomized trial comparing oxygen administration during nasal flexible bronchoscopy: oral vs nasal delivery. Chest.

[16] Miyagi K, Haranaga S, Higa F, Tateyama M, Fujita J (2014). Implementation of bronchoalveolar lavage using a high-flow nasal cannula in five cases of acute respiratory failure. Respir Investig.

[17] La Combe B, Messika J, Labbé V, Razazi K, Maitre B, Sztrymf B (2016). High-flow nasal oxygen for bronchoalveolar lavage in acute respiratory failure patients. Eur Respir J.

[18] Longhini F, Pelaia C, Garofalo E, Bruni A, Placida R, Iaquinta C (2021). High-flow nasal cannula oxygen therapy for outpatients undergoing flexible bronchoscopy: a randomised controlled trial. Thorax.

[19] Chung SM, Choi JW, Lee YS, Choi JH, Oh JY, Min KH (2019). Clinical effectiveness of high-flow nasal cannula in hypoxaemic patients during bronchoscopic procedures. Tuberc Respir Dis.

[20] Service JA, Bain JS, Gardner CP, McNarry AF (2019). Prospective experience of high-flow nasal oxygen during bronchoscopy in 182 patients: a feasibility study. J Bronchol Interv Pulmonol.

[21] Ben-Menachem E, McKenzie J, O’Sullivan C, Havryk AP (2020). High-flow nasal oxygen versus standard oxygen during flexible bronchoscopy in lung transplant patients: a randomized controlled trial. J Bronchol Interv Pulmonol.

[22] Lucangelo U, Vassallo FG, Marras E, Ferluga M, Beziza E, Comuzzi L (2012). High-flow nasal interface improves oxygenation in patients undergoing bronchoscopy. Crit Care Res Pract..

[23] Irfan M, Ahmed M, Breen D (2021). Assessment of high flow nasal cannula oxygenation in endobronchial ultrasound bronchoscopy: a randomized controlled trial. J Bronchol Interv Pulmonol.

[24] Douglas N, Ng I, Nazeem F, Lee K, Mezzavia P, Krieser R (2018). A randomised controlled trial comparing high-flow nasal oxygen with standard management for conscious sedation during bronchoscopy. Anaesthesia.

[25] Kim EJ, Jung CY, Kim KC (2018). Effectiveness and safety of high-flow nasal cannula oxygen delivery during bronchoalveolar lavage in acute respiratory failure patients. Tuberc Respir Dis.

[26] Maitre B, Jaber S, Maggiore SM, Bergot E, Richard JC, Bakthiari H (2000). Continuous positive airway pressure during fiberoptic bronchoscopy in hypoxemic patients. A randomized double-blind study using a new device. Am J Respir Crit Care Med.

[27] Antonelli M, Conti G, Rocco M, Arcangeli A, Cavaliere F, Proietti R (2002). Noninvasive positive-pressure ventilation vs conventional oxygen supplementation in hypoxemic patients undergoing diagnostic bronchoscopy. Chest.

[28] Baumann HJ, Klose H, Simon M, Ghadban T, Braune SA, Hennigs JK (2011). Fiber optic bronchoscopy in patients with acute hypoxemic respiratory failure requiring noninvasive ventilation—a feasibility study. Crit Care.

[29] Korkmaz Ekren P, Basarik Aydogan B, Gurgun A, Tasbakan MS, Bacakoglu F, Nava S (2016). Can fiberoptic bronchoscopy be applied to critically ill patients treated with noninvasive ventilation for acute respiratory distress syndrome? Prospective observational study. BMC Pulm Med.

[30] Chiner E, Sancho-Chust JN, Llombart M, Senent C, Camarasa A, Signes-Costa J (2010). Fiberoptic bronchoscopy during nasal non-invasive ventilation in acute respiratory failure. Respir Int Rev Thorac Dis.

[31] Heunks LMA, de Bruin CJR, van der Hoeven JG, van der Heijden HFM (2010). Non-invasive mechanical ventilation for diagnostic bronchoscopy using a new face mask: an observational feasibility study. Intensive Care Med.

[32] Antonelli M, Pennisi MA, Conti G, Bello G, Maggiore SM, Michetti V (2003). Fiberoptic bronchoscopy during noninvasive positive pressure ventilation delivered by helmet. Intensive Care Med.

[33] Agarwal R, Khan A, Aggarwal AN, Gupta D (2012). Bronchoscopic lung biopsy using noninvasive ventilatory support: case series and review of literature of NIV-assisted bronchoscopy. Respir Care.

[34] Chen X, Zhou Y, Yu H, Peng Y, Xia L, Liu N (2020). Feasibility analysis of flexible bronchoscopy in conjunction with noninvasive ventilation for therapy of hypoxemic patients with Central Airway Obstruction: a retrospective study. PeerJ.

[35] Da Conceiçao M, Genco G, Favier JC, Bidallier I, Pitti R (2000). Fiberoptic bronchoscopy during noninvasive positive-pressure ventilation in patients with chronic obstructive lung disease with hypoxemia and hypercapnia. Ann Francaises D’anesthesie Et De Reanimation.

[36] Mohamed AS, El-Sharawy DE (2018). Noninvasive ventilation with add-on fiberoptic bronchoscopy in patients with chronic obstructive pulmonary disease. Egypt J Chest Dis Tuberc.

[37] Scala R, Naldi M, Maccari U (2010). Early fiberoptic bronchoscopy during non-invasive ventilation in patients with decompensated chronic obstructive pulmonary disease due to community-acquired-pneumonia. Crit Care.

[38] Simon M, Braune S, Frings D, Wiontzek A-K, Klose H, Kluge S (2014). High-flow nasal cannula oxygen versus non-invasive ventilation in patients with acute hypoxaemic respiratory failure undergoing flexible bronchoscopy-a prospective randomised trial. Crit Care.

[39] Saksitthichok B, Petnak T, So-Ngern A, Boonsarngsuk V (2019). A prospective randomized comparative study of high-flow nasal cannula oxygen and non-invasive ventilation in hypoxemic patients undergoing diagnostic flexible bronchoscopy. J Thorac Dis.

[40] Lindholm CE, Ollman B, Snyder JV, Millen EG, Grenvik A (1978). Cardiorespiratory effects of flexible fiberoptic bronchoscopy in critically ill patients. Chest.

[41] Matsushima Y, Jones RL, King EG, Moysa G, Alton JD (1984). Alterations in pulmonary mechanics and gas exchange during routine fiberoptic bronchoscopy. Chest.

[42] Miller EJ (1979). Hypoxemia during fiberoptic bronchoscopy. Chest.

[43] Lundgren R, Häggmark S, Reiz S (1982). Hemodynamic effects of flexible fiberoptic bronchoscopy performed under topical anesthesia. Chest.

[44] Davies L, Mister R, Spence DP, Calverley PM, Earis JE, Pearson MG (1997). Cardiovascular consequences of fibreoptic bronchoscopy. Eur Respir J.

[45] Albertini R, Harrel JH, Moser KM (1974). Letter: hypoxemia during fiberoptic bronchoscopy. Chest.

[46] Pisani L, Astuto M, Prediletto I, Longhini F (2019). High flow through nasal cannula in exacerbated COPD patients: a systematic review. Pulmonology.

[47] Cortegiani A, Longhini F, Carlucci A, Scala R, Groff P, Bruni A (2019). High-flow nasal therapy versus noninvasive ventilation in COPD patients with mild-to-moderate hypercapnic acute respiratory failure: study protocol for a noninferiority randomized clinical trial. Trials.

[48] Garofalo E, Bruni A, Pelaia C, Cammarota G, Murabito P, Biamonte E (2019). Evaluation of a new interface combining high-flow nasal cannula and CPAP. Respir Care.

[49] Renda T, Corrado A, Iskandar G, Pelaia G, Abdalla K, Navalesi P (2018). High-flow nasal oxygen therapy in intensive care and anaesthesia. Br J Anaesth.

[50] Cammarota G, Vaschetto R, Turucz E, Dellapiazza F, Colombo D, Blando C (2011). Influence of lung collapse distribution on the physiologic response to recruitment maneuvers during noninvasive continuous positive airway pressure. Intensive Care Med.

[51] Lindner KH, Lotz P, Ahnefeld FW (1987). Continuous positive airway pressure effect on functional residual capacity, vital capacity and its subdivisions. Chest.

[52] Longhini F, Colombo D, Pisani L, Idone F, Chun P, Doorduin J (2017). Efficacy of ventilator waveform observation for detection of patient-ventilator asynchrony during NIV: a multicentre study. ERJ Open Res.

[53] Cammarota G, Olivieri C, Costa R, Vaschetto R, Colombo D, Turucz E (2011). Noninvasive ventilation through a helmet in postextubation hypoxemic patients: physiologic comparison between neurally adjusted ventilatory assist and pressure support ventilation. Intensive Care Med.

[54] Longhini F, Pan C, Xie J, Cammarota G, Bruni A, Garofalo E (2017). New setting of neurally adjusted ventilatory assist for noninvasive ventilation by facial mask: a physiologic study. Crit Care.

[55] Bruni A, Garofalo E, Pelaia C, Messina A, Cammarota G, Murabito P (2019). Patient-ventilator asynchrony in adult critically ill patients. Minerva Anestesiol.

[56] Eastwood PR, Platt PR, Shepherd K, Maddison K, Hillman DR (2005). Collapsibility of the upper airway at different concentrations of propofol anesthesia. Anesthesiology.

[57] Shteamer JW, Dedhia RC (2017). Sedative choice in drug-induced sleep endoscopy: a neuropharmacology-based review. Laryngoscope.

[58] Costa R, Navalesi P, Cammarota G, Longhini F, Spinazzola G, Cipriani F (2017). Remifentanil effects on respiratory drive and timing during pressure support ventilation and neurally adjusted ventilatory assist. Respir Physiol Neurobiol.

[59] Vaschetto R, Cammarota G, Colombo D, Longhini F, Grossi F, Giovanniello A (2014). Effects of propofol on patient-ventilator synchrony and interaction during pressure support ventilation and neurally adjusted ventilatory assist. Crit Care Med.

[60] Sampsonas F, Kakoullis L, Karampitsakos T, Papaioannou O, Katsaras M, Papachristodoulou E (2021). Bronchoscopy during the COVID-19 pandemic: effect on current practices and strategies to reduce procedure-associated transmission. Expert Rev Respir Med.

[61] Guan L, Zhou L, Zhang J, Peng W, Chen R (2020). More awareness is needed for severe acute respiratory syndrome coronavirus 2019 transmission through exhaled air during non-invasive respiratory support: experience from China. Eur Respir J.

[62] Hui DS, Chow BK, Lo T, Ng SS, Ko FW, Gin T (2015). Exhaled air dispersion during noninvasive ventilation via helmets and a total facemask. Chest.

[63] Hui DS, Chow BK, Lo T, Tsang OTY, Ko FW, Ng SS (2019). Exhaled air dispersion during high-flow nasal cannula therapy versus CPAP via different masks. Eur Respir J.

