# Supplementary material for: Oxygenation strategies during flexible bronchoscopy: a review of the literature
Source: Respir Res. 2021 Sep 25;22:253. doi: 10.1186/s12931-021-01846-1 (PMC8464093; doi:10.1186/s12931-021-01846-1)

**ADDITIONAL MATERIAL**

**OXYGENATION STRATEGIES DURING FLEXIBLE BRONCHOSCOPY: A REVIEW OF THE LITERATURE**

Corrado Pelaia, MD; Andrea Bruni, MD; Eugenio Garofalo, MD; Serena Rovida, MD; Eugenio Arrighi, MD; Gianmaria Cammarota, MD, PhD; Paolo Navalesi, MD, FERS^;^ Girolamo Pelaia, MD; Federico Longhini, MD.

**SEARCH STRATEGY**

Search strategies for the three queried scientific databases are the following:

**- MEDLINE**

((“Bronchoscopy” [MeSH] OR “Bronchoscopies” [MeSH] OR “Bronchoscopic Surgical Procedures” [MeSH] OR “Bronchoscopic Surgical Procedure” [MeSH] OR “Surgical Procedure, Bronchoscopic” [MeSH] OR “Surgical Procedures, Bronchoscopic” [MeSH] OR “Bronchoscopic Surgery” [MeSH] OR “Bronchoscopic Surgeries” [MeSH] OR “Surgeries, Bronchoscopic” [MeSH] OR “Surgery, Bronchoscopic” [MeSH] OR “Bronchoscopy” [All fields] OR “Bronchoscopies” [All fields] OR “Bronchoscopic Surgical Procedures” [All fields] OR “Bronchoscopic Surgical Procedure” [All fields] OR “Surgical Procedure, Bronchoscopic” [All fields] OR “Surgical Procedures, Bronchoscopic” [All fields] OR “Bronchoscopic Surgery” [All fields] OR “Bronchoscopic Surgeries” [All fields] OR “Surgeries, Bronchoscopic” [All fields] OR “Surgery, Bronchoscopic” [All fields] OR “Bronchial endoscopy” [All fileds]) AND (“Oxygen” [MeSH] OR “Continuous Positive Airway Pressure” [MeSH] OR “CPAP Ventilation” [MeSH] OR “Ventilation, CPAP” [MeSH] OR “Biphasic Continuous Positive Airway Pressure” [MeSH] OR “Bilevel Continuous Positive Airway Pressure” [MeSH] OR “Nasal Continuous Positive Airway Pressure” [MeSH] OR “nCPAP Ventilation” [MeSH] OR “Ventilation, nCPAP” [MeSH] OR “Airway Pressure Release Ventilation” [MeSH] OR “APRV Ventilation Mode” [MeSH] OR “APRV Ventilation Modes” [MeSH] OR “Ventilation Mode, APRV” [MeSH] OR “Ventilation Modes, APRV” [MeSH] OR “Noninvasive ventilation” [MeSH] OR “Noninvasive Ventilations” [MeSH] OR “Ventilation, Noninvasive” [MeSH] OR “Ventilations, Noninvasive” [MeSH] OR “Non-Invasive Ventilation” [MeSH] OR “Non-Invasive Ventilations” [MeSH] OR “Ventilation, Non-Invasive” [MeSH] OR “Ventilations, Non-Invasive” [MeSH] OR “Non Invasive Ventilation” [MeSH] OR “Non Invasive Ventilations” [MeSH] OR “Ventilation, Non Invasive” [MeSH] OR “Ventilations, Non Invasive” [MeSH] OR “Oxygen” [All fields] OR “conventional oxygen therapy” [All fields] OR “oxygen therapy” [All fields] OR “oxygenation” [All fields] OR “Continuous Positive Airway Pressure” [All fields] OR “CPAP Ventilation” [All fields] OR “Ventilation, CPAP” [All fields] OR “Biphasic Continuous Positive Airway Pressure” [All fields] OR “Bilevel Continuous Positive Airway Pressure” [All fields] OR “Nasal Continuous Positive Airway Pressure” [All fields] OR “nCPAP Ventilation” [All fields] OR “Ventilation, nCPAP” [All fields] OR “Airway Pressure Release Ventilation” [All fields] OR “APRV Ventilation Mode” [All fields] OR “APRV Ventilation Modes” [All fields] OR “Ventilation Mode, APRV” [All fields] OR “Ventilation Modes, APRV” [All fields] OR “Noninvasive ventilation” [All fields] OR “Noninvasive Ventilations” [All fields] OR “Ventilation, Noninvasive” [All fields] OR “Ventilations, Noninvasive” [All fields] OR “Non-Invasive Ventilation” [All fields] OR “Non-Invasive Ventilations” [All fields] OR “Ventilation, Non-Invasive” [All fields] OR “Ventilations, Non-Invasive” [All fields] OR “Non Invasive Ventilation” [All fields] OR “Non Invasive Ventilations” [All fields] OR “Ventilation, Non Invasive” [All fields] OR “Ventilations, Non Invasive” [All fields] OR “NIV” [All fields] OR "high flow nasal oxygen" [All Fields] OR "high-flow nasal oxygen" [All Fields] OR "high flow nasal cannula" [All Fields] OR “high-flow nasal cannula" [All Fields] OR "high flow oxygen" [All Fields] OR "high-flow oxygen" [All Fields] OR “High-Flow Oxygen Therapy” [All Fields]))

**- EMBASE**

(("Bronchoscopy" OR "Bronchoscopies" OR "Bronchoscopic Surgical Procedures" OR "Bronchoscopic Surgical Procedure" OR "Surgical Procedure, Bronchoscopic" OR "Surgical Procedures, Bronchoscopic" OR "Bronchoscopic Surgery" OR "Bronchoscopic Surgeries" OR "Surgeries, Bronchoscopic" OR "Surgery, Bronchoscopic" OR "Bronchial endoscopy") AND ("conventional oxygen therapy" OR "oxygen therapy" OR "oxygenation" OR "Continuous Positive Airway Pressure" OR "CPAP Ventilation" OR "Ventilation, CPAP" OR "Biphasic Continuous Positive Airway Pressure" OR "Bilevel Continuous Positive Airway Pressure" OR "Nasal Continuous Positive Airway Pressure" OR "nCPAP Ventilation" OR "Ventilation, nCPAP" OR "Airway Pressure Release Ventilation" OR "APRV Ventilation Mode" OR "APRV Ventilation Modes" OR "Ventilation Mode, APRV" OR "Ventilation Modes, APRV" OR "Noninvasive ventilation" OR "Noninvasive Ventilations" OR "Ventilation, Noninvasive" OR "Ventilations, Noninvasive" OR "Non-Invasive Ventilation" OR "Non-Invasive Ventilations" OR "Ventilation, Non-Invasive" OR "Ventilations, Non-Invasive" OR "Non Invasive Ventilation" OR "Non Invasive Ventilations" OR "Ventilation, Non Invasive" OR "Ventilations, Non Invasive" OR "NIV" OR "high flow nasal oxygen" OR "high-flow nasal oxygen" OR "high flow nasal cannula" OR "high-flow nasal cannula" OR "high flow oxygen" OR "high-flow oxygen" OR "High-Flow Oxygen Therapy" )) AND ('article'/it OR 'article in press'/it) AND ([adult]/lim OR [aged]/lim OR [middle aged]/lim OR [very elderly]/lim OR [young adult]/lim)

**- Scopus**

TITLE-ABS-KEY (("Bronchoscopy" OR "Bronchoscopies" OR "Bronchoscopic Surgical Procedures" OR "Bronchoscopic Surgical Procedure" OR "Surgical Procedure, Bronchoscopic" OR "Surgical Procedures, Bronchoscopic" OR "Bronchoscopic Surgery" OR "Bronchoscopic Surgeries" OR "Surgeries, Bronchoscopic" OR "Surgery, Bronchoscopic" OR "Bronchial endoscopy") AND ("conventional oxygen therapy" OR "oxygen therapy" OR "oxygenation" OR "Continuous Positive Airway Pressure" OR "CPAP Ventilation" OR "Ventilation, CPAP" OR "Biphasic Continuous Positive Airway Pressure" OR "Bilevel Continuous Positive Airway Pressure" OR "Nasal Continuous Positive Airway Pressure" OR "nCPAP Ventilation" OR "Ventilation, nCPAP" OR "Airway Pressure Release Ventilation" OR "APRV Ventilation Mode" OR "APRV Ventilation Modes" OR "Ventilation Mode, APRV" OR "Ventilation Modes, APRV" OR "Noninvasive ventilation" OR "Noninvasive Ventilations" OR "Ventilation, Noninvasive" OR "Ventilations, Noninvasive" OR "Non-Invasive Ventilation" OR "Non-Invasive Ventilations" OR "Ventilation, Non-Invasive" OR "Ventilations, Non-Invasive" OR "Non Invasive Ventilation" OR "Non Invasive Ventilations" OR "Ventilation, Non Invasive" OR "Ventilations, Non Invasive" OR "NIV" OR "high flow nasal oxygen" OR "high-flow nasal oxygen" OR "high flow nasal cannula" OR "high-flow nasal cannula" OR "high flow oxygen" OR "high-flow oxygen" OR "High-Flow Oxygen Therapy" )) AND (LIMIT-TO ( DOCTYPE , "ar" ) )

**Figure S1 – Funnel plot for HFNC versus COT with respect to the lowest saturation during FOB**


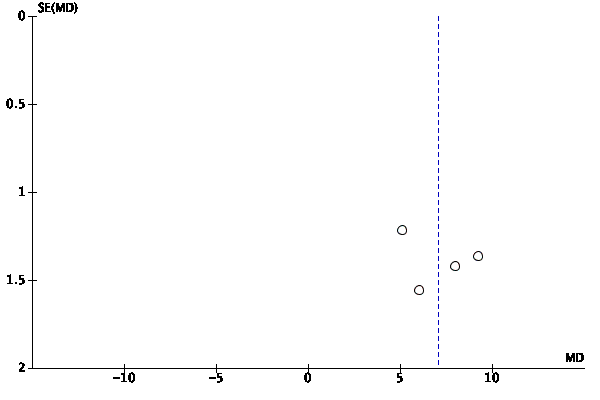


**Figure S2 – Funnel plot for HFNC versus COT with respect to the episodes of desaturation**


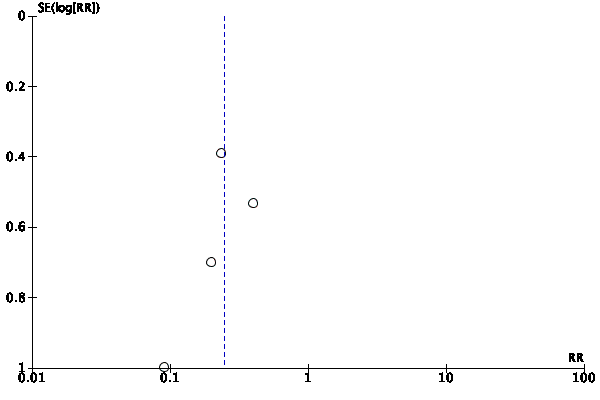


**Figure S3 – Funnel plot for HFNC versus NIV with respect to the lowest saturation during FOB**


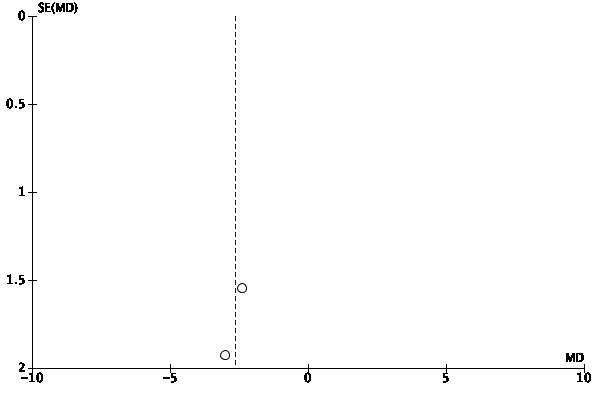


**Figure S4 – Funnel plot for HFNC versus NIV with respect to the episodes of desaturation**


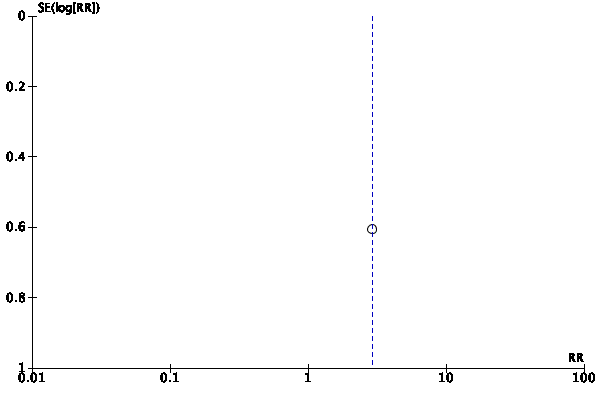

Supplement: Supplementary file 1 — Additional file 1: Figure S1. Funnel plot for HFNC versus COT with respect to the lowest saturation during FOB. Figure S2. Funnel plot for HFNC versus COT with respect to the episodes of desaturation. Figure S3. Funnel plot for HFNC versus NIV with respect to the lowest saturation during FOB. Figure S4. Funnel plot for HFNC versus NIV with respect to the episodes of desaturation. [file 12931_2021_1846_MOESM1_ESM.docx]
